# Supplementary material for: Structured matching models in multimodal information fusion: An optimized Kuhn-Munkres algorithm
Source: PLoS One. 2025 Nov 21;20(11):e0326662. doi: 10.1371/journal.pone.0326662 (PMC12637911; doi:10.1371/journal.pone.0326662)
Supplement: S1 Appendix — (DOCX) [file pone.0326662.s001.docx]

Appendix S1:

Pseudo-code of the Improved Kuhn-Munkres Algorithm (Integrating Dynamic Weighting and Correlation Constraints)

Algorithm: Multimodal Kuhn-Munkres with Dynamic Weights and Correlation Constraints

Input:

- Feature sets {F₁, F₂, ..., F_k} for k modalities

- Similarity functions {f₁(u,v), f₂(u,v), ..., f_k(u,v)}

- Correlation matrix C ∈ ℝ^(k×k)

- Correlation threshold ϵ

- Maximum iterations Tmax

- Weight update interval T_update

Output:

- Optimal matching M ⊆ (U × V)

1: Initialize weight vector α = [1/k, 1/k, ..., 1/k]

2: Initialize iteration counter t ← 0

3: repeat

4: for each candidate pair (u,v) in bipartite graph G(U,V):

5: Compute weighted similarity:

w(u,v) = Σᵢ αᵢ · fᵢ(u,v)

6: if C(i,j)·w(u,v) < ϵ then

7: Set w(u,v) ← 0 // Remove unreliable match

8: end if

9: end for

10: Solve maximum weight matching on G using Kuhn-Munkres

11: if t mod T_update == 0 then

12: for each modality i = 1 to k:

13: Compute Var(fᵢ) over matched pairs

14: end for

15: Normalize αᵢ ← Var(fᵢ) / Σⱼ Var(fⱼ)

16: end if

17: t ← t + 1

18: until convergence or t ≥ Tmax

19: return final matching M
