# Supplementary material for: Structured matching models in multimodal information fusion: An optimized Kuhn-Munkres algorithm
Source: PLoS One. 2025 Nov 21;20(11):e0326662. doi: 10.1371/journal.pone.0326662 (PMC12637911; doi:10.1371/journal.pone.0326662)
Supplement: Attachment — Submitted filename: manuscript.docx [file pone.0326662.s003.docx]

**Information Integration Model in Interaction Design under Computer Multimodal Collaboration: The Integrated Application of Kuhn-Munkres Algorithm**

Jinxia Wang1*, Qingnan Ji1

1, Art design College of Shaanxi Fashion Engineering university, Xi'an City 712046, China

*Corrseponding author: Jinxia Wang, E-mail: 1754354519@qq.com

**Abstract**

In modern multimodal interaction design, how to effectively integrate information from various modalities such as speech, vision, and text has become a significant challenge. These modalities exhibit issues such as heterogeneity, temporality, and differences in data volume, which lead to problems such as inaccurate matching, low computational efficiency, and insufficient user experience during the information integration process. This study aims to improve the efficiency and accuracy of information integration in multimodal interaction environments. Firstly, this study utilizes the publicly available Carnegie Mellon University Multimodal Opinion Sentiment Intensity (CMU-MOSI) and Interactive Emotional Dyadic Motion Capture (IEMOCAP) databases to obtain speech, vision, and text data required for multimodal interaction design. The data undergoes preprocessing, including noise removal, feature extraction (e.g., Mel Frequency Cepstral Coefficients, keypoint detection), and temporal alignment. Secondly, an improved Kuhn-Munkres algorithm is proposed, which extends the original bipartite graph matching to a weighted multimodal matching model. The algorithm's performance is optimized by dynamically adjusting weight coefficients (based on modal feature importance scores). Additionally, a cross-modal correlation matrix is incorporated as a constraint to enhance the robustness of the matching process. Finally, the practical performance of the improved algorithm in multimodal information integration is verified through information matching efficiency tests and user interaction satisfaction surveys. Experimental results show that the improved Kuhn-Munkres algorithm increases multimodal information matching accuracy by 28.2% compared to the baseline method. It also improves integration efficiency by 18.7% and significantly reduces computational complexity, with an average computation time reduction of 15.4%. The user interaction satisfaction survey results reveal a 19.5% improvement in user experience ratings, further confirming the model's practical applicability. This study presents an innovative optimization method for multimodal information integration. It offers significant theoretical value and broad potential applications in intelligent interaction design and human-computer collaboration. These contributions help advance the development of multimodal interaction systems.

**Keywords:** Multimodal Interaction; Information Integration Model; Human-Computer Interaction; Kuhn-Munkres Algorithm.

Introduction

Multimodal interaction design, as an important technological approach, has garnered widespread attention in the fields of artificial intelligence and human-computer interaction. With the rapid development of speech recognition, computer vision, and natural language processing technologies, multimodal technology can effectively integrate heterogeneous modalities such as speech, vision, and text. This integration provides richer interaction experiences for intelligent devices and systems [1]. For example, multimodal technology is widely applied in areas like emotion recognition, virtual assistants, and intelligent monitoring. It offers more intelligent services by comprehensively analyzing information from different modalities [2]. Compared to single-modality approaches, multimodal interaction design has the advantages of information complementarity and strong robustness, enabling better performance in complex scenarios [3].

In the core process of multimodal information processing, information integration is the key factor determining the system’s performance. Multimodal information integration aims to effectively match and fuse data from modalities such as speech, vision, and text, thereby generating higher-quality integrated information [4]. However, there are challenges in the information integration process due to differences in feature dimensions, time scales, and presentation formats across modalities. These differences often create a bottleneck that restricts the further development of multimodal technologies [5]. Therefore, studying how to efficiently integrate multimodal information in complex scenarios is of great significance for improving the intelligence level of human-computer interaction.

Despite significant progress in multimodal interaction technology in recent years, there remain many challenges in the multimodal information integration process. Firstly, the heterogeneity of modality data makes it difficult to directly fuse features from different modalities [6]. Secondly, the temporal issues of modality data present another major challenge, as different modalities often experience asynchronous timing, particularly in dynamic scenarios where this problem becomes more pronounced [7]. Furthermore, many existing information matching methods have low efficiency and cannot meet the requirements in large-scale data processing or real-time applications. Especially in complex tasks, traditional matching algorithms often require substantial computational resources, and the matching results may not be ideal [8]. More importantly, current methods also exhibit shortcomings in terms of user experience. Traditional approaches lack flexibility in multimodal information matching and struggle to adapt to the dynamic changes in user needs. This inaccuracy in matching and response delays directly impact user satisfaction with multimodal interaction systems.

To address these issues in multimodal information integration, this study proposes an improved method based on the Kuhn-Munkres algorithm. By introducing a dynamic weight adjustment mechanism and inter-modal correlation constraints, the method optimizes the multimodal matching performance of the algorithm, significantly improving the efficiency and robustness of information integration. This study, based on publicly available datasets Carnegie Mellon University Multimodal Opinion Sentiment Intensity Database (CMU-MOSI) and Interactive Emotional Dyadic Motion Capture Database (IEMOCAP), conducted experimental validation of multimodal interaction tasks and achieved promising results.

## Literature review

Under the advancement of deep learning, significant progress has been made in the methods of multimodal information integration. Liu et al. (2024) adopted a multimodal Transformer architecture, utilizing a cross-modal attention mechanism to integrate speech, text, and visual modalities, greatly improving the accuracy of emotion recognition [9]. Additionally, the development of data resources in multimodal research has also played a crucial supporting role. The core idea of the Kuhn-Munkres algorithm is to find the optimal matching in a bipartite graph. It achieves this through labeling and path extension. This algorithm is widely used in fields such as task allocation and resource scheduling [10]. For example, in traffic systems, the Kuhn-Munkres algorithm is applied to optimize the matching between vehicles and orders to maximize efficiency [11].

However, the direct application of the Kuhn-Munkres algorithm in multimodal information matching faces several challenges. Existing methods typically assume that the matching weights between modalities are fixed. In practice, multimodal information is often heterogeneous, and the distribution of modality feature weights may dynamically change depending on the scenario [12, 13]. The algorithm's efficiency decreases when processing large-scale, high-dimensional data. This issue is particularly noticeable when additional constraints are introduced in complex multimodal tasks, leading to a significant increase in computational cost [14]. To address these challenges, researchers have proposed several improvements. For example, heuristic search-based acceleration methods [15] have been suggested. However, these improvements mainly focus on traditional fixed-weight scenarios and provide limited support for multimodal weighted matching.

In summary, significant progress has been made in the research on multimodal information integration methods. The combination of deep learning and graph models provides new solutions for multimodal technologies, and the application of the Kuhn-Munkres algorithm in information matching offers theoretical support for task optimization. However, the heterogeneity of multimodal information, dynamic weight distribution, and the introduction of complex constraints remain unsolved challenges.

Materials and methods

## Data Sources and Preprocessing

This study selected CMU-MOSI and IEMOCAP as the data sources to ensure the scientific and widely applicable construction and validation of the multimodal information integration model. The CMU-MOSI dataset contains data from three modalities—speech, vision, and text—primarily used for multimodal sentiment analysis. It includes annotations of sentiment intensity in video comments, making it suitable for studying modality coordination mechanisms and improvements in weighted matching methods. The IEMOCAP dataset also contains speech, vision, and text modalities, but its data come from human emotional expression in conversational scenarios, featuring authentic dynamic interaction characteristics. This provides a rich testing ground for validating the improved Kuhn-Munkres algorithm in multimodal data matching.

The complementarity of the two datasets enables this study to test the algorithm's performance in both sentiment intensity analysis and dynamic interaction, ensuring that the results are widely applicable. The speech modality in the dataset is stored in audio file format, the visual modality provides a sequence of facial expression frames, and the text modality includes manually annotated transcribed text [16, 17]. All of these data types have been rigorously annotated and verified, supporting the study needs for modality feature importance scoring and weight allocation.

To ensure the consistency and effectiveness of multimodal data in information integration, the following data preprocessing steps were adopted: noise removal, feature extraction, and multimodal temporal alignment. The denoising process for speech signals uses Short-Time Fourier Transform (STFT) and Spectral Subtraction. This process improves the signal-to-noise ratio by removing environmental noise and low-frequency interference from the speech data [18, 19]. Specifically, let the audio signal be *x(t)* and its spectrum be *X(f)*. By estimating the noise spectrum *N(f)*, the denoised spectrum can be calculated, as shown in Equation (1):

$S\left( f \right)=X\left( f \right)-N\left( f \right),if X(f)>N(f);0,otherwise$	(1)

The denoising of visual data is performed using the Median Filtering technique to reduce random noise caused by lighting or sensor issues in the frame sequence [20]. Additionally, optical flow methods are applied to detect key region changes between frames, further improving the robustness of facial expression feature extraction.

For the speech modality, Mel-Frequency Cepstral Coefficients (MFCC) are used for feature extraction. MFCC imitates human auditory perception by mapping spectral energy onto the Mel scale. The Mel frequency *m* corresponding to a frequency *f* is expressed as shown in Equation (2):

$m=2595\cdot{log}_{10}\boldsymbol{(}1+f/700)$	(2)

By extracting the MFCC feature vector, the short-term spectral characteristics of the speech signal can be efficiently captured. For the visual modality, feature extraction is based on keypoint detection technology using the Dlib library to recognize facial keypoints. From these keypoints, facial action coding system (FACS) related features are extracted [21]. For text modality, feature extraction is achieved through Word Embedding technology. The Bidirectional Encoder Representations from Transformers (BERT) model is used to transform text into high-dimensional vector representations, capturing contextual semantic information [22].

Temporal alignment of multimodal data is a key step in achieving information integration between modalities. In this study, the Dynamic Time Warping (DTW) algorithm is used for temporal alignment. DTW minimizes the distance between time series of different modalities, addressing the issue of inconsistent step sizes across modalities. Let the two modality time series be *A*= {*a*_1_, *a*_2_, …, *a_n_*} and *B*= {*b*_1_, *b*_2_, …, *b_m_*}, the DTW distance *D*(*i*, *j*) is given by Equation (3):

$D(i,j)=\parallel a_{i}-b_{j}\parallel+min\{D(i-1,j),D(i,j-1),D(i-1,j-1)\}$	(3)

∥*a_i_*−*b_j_*∥ is the Euclidean distance between the features of the two modalities, and the final alignment path is derived using dynamic programming.

Through the above-mentioned denoising, feature extraction, and temporal alignment steps, this study provides high-quality data input for the subsequent research and validation of multimodal information matching algorithms.

## Improvement of the Kuhn-Munkres Algorithm

The Kuhn-Munkres algorithm is a classical maximum-weight bipartite graph matching algorithm. Its primary goal is to find the optimal matching for the vertices in a bipartite graph, such that the total weight is maximized, as shown in Fig. 1.

**Fig 1.** Structure of the Kuhn-Munkres Algorithm

For a bipartite graph *G*= (*U*, *V*, *E*), where *U* and *V* are two sets of vertices and *E* is the set of edges connecting the vertices, each edge *e(u,v)* has a weight *w(u,v)*. The algorithm traverses the vertices and edges to find the optimal allocation that maximizes the total matching weight. The core of the algorithm lies in constructing an auxiliary graph, where the vertex labeling and path extension iteratively optimize the matching results. Let *L*(*u*) and *R*(*v*) be the labels of vertices *u*∈*U* and *v*∈*V*, respectively. For any *u, v*, it satisfies:

In each iteration, the labels are adjusted so that the current matching scheme converges toward optimality. The time complexity of the Kuhn-Munkres algorithm is *O*(∣*U*∣^2^⋅∣*V*∣), which provides high efficiency for bipartite graph matching problems [23, 24]. In the context of multimodal interaction design and information matching, this study improves the Kuhn-Munkres algorithm to extend its applicability and matching performance.

Traditional Kuhn-Munkres algorithms primarily handle bipartite graph matching problems and do not support the weighted allocation of multimodal information [25, 26]. This study introduces a multimodal weighting mechanism. It treats multimodal features as attributes of the vertices. The edge weights are adjusted based on the importance of the modality features during the matching process. Let the multimodal feature set be {*M*_1_, *M*_2_, …, *M_k_*}. The edge weight is defined as shown in Equation (4):

$w(u,v)=\sum_{i=1}^{k} \alpha_{i}\cdot f_{i}(u,v)$	(4)

*α_i_* is the weight of modality *M_i_*, and *fi*(*u*,*v*) is the similarity function between modality features. By reasonably allocating the weight *α_i_*, the model can more precisely reflect the integration needs of multimodal information.

To further enhance the algorithm's adaptability in different scenarios, this study designs a dynamic weight adjustment mechanism, which adjusts the value of *α_i_* based on the importance score of modality features. The importance score of modality features is determined by their discriminability and confidence [27, 28]. The specific calculation is given by Equation (5):

$\alpha_{i}=\frac{\mathrm{Var}(f_{i})}{\sum_{j=1}^{k} \mathrm{Var}(f_{j})}$	(5)

Var(*f_i_*) is the variance of modality *M_i_*'s feature, representing its contribution to the matching result. The dynamic adjustment mechanism allows for real-time weight adjustment for each modality based on the distribution of multimodal data, thus improving the matching accuracy of the algorithm.

There is often some correlation between multimodal data, such as how speech and facial expressions jointly convey emotion [29, 30]. To fully utilize this correlation in matching, this study introduces an inter-modal cross-correlation matrix *C* as a constraint. During the matching process, the matching results must satisfy Equation (6):

$C(i,j)\cdot w(u,v)>\epsilon$	(6)

$C(i,j)$ is the cross-correlation between modalities *M_i_* and *M_j_*, and $\epsilon$ is a predefined correlation threshold. By introducing this constraint, the algorithm effectively eliminates inconsistent matching results between modalities and enhances the robustness of the matching model.

## Experimental Design

To comprehensively evaluate the practical performance of the improved Kuhn-Munkres algorithm in multimodal interaction design, two sets of experiments were designed: Information Matching Efficiency Testing and User Interaction Satisfaction Survey.

This study has been approved by the Ethics Approval Committee of Shaanxi Institute of Fashion Engineering and obtained the written informed consent of relevant participants. All studies are conducted in accordance with the relevant regulations of the relevant departments. The experiment began in January 2024 and ended in August 2024.

In order to compare the performance of the improved algorithm comprehensively, this study selected five state-of-the-art multimodal data matching algorithms as baseline methods:

Traditional Kuhn-Munkres Algorithm: Used as the baseline for the original algorithm to validate the performance improvements of the proposed method.

Deep Canonical Correlation Analysis (DeepCCA): A deep learning-based multimodal correlation analysis method, used for comparison in terms of correlation modeling capabilities.

Multimodal Tensor Fusion (MMTF): An algorithm for multimodal feature fusion based on tensor decomposition, used for comparison in multimodal feature fusion efficiency.

Graph-Matching Network (GMN): A graph neural network-based multimodal graph matching algorithm, used to compare the proposed method's advantage in graph structure modeling.

Cross-Attention Transformer (CAT): A matching method based on cross-modal attention mechanisms, used for comparing the effect of dynamic weight adjustment in modality collaborative optimization.

To fully evaluate the performance of each algorithm, five comparison metrics were designed, covering aspects such as matching accuracy, efficiency, and user experience:

Matching Accuracy: Measures the accuracy of the algorithm in multimodal information matching tasks, calculated as shown in Equation (7):

$\mathrm{Accuracy}=\frac{\text{Number of correct matches}}{\text{Total number of matches}}\times100\%$	(7)

Matching Time: Measures the time required for the algorithm to complete a full matching task, expressed in seconds, serving as an indicator of its computational efficiency.

Computational Complexity: Assesses the algorithm's theoretical computational complexity using Big-O notation to characterize its time complexity.

Information Integration Efficiency: Quantifies the overall effectiveness of information integration by incorporating both matching time and accuracy, yielding a comprehensive efficiency score.

User Satisfaction Score: Derives the average user experience rating from a standardized interaction satisfaction survey, with scores ranging from 1 to 10.

The hyperparameter settings for this algorithm are shown in Table 1.

**Table 1.** Hyperparameter Settings

| **Hyperparameter Name** | **Range of Values** | **Final Value** |
| --- | --- | --- |
| **Initial Modality Feature Weight** | [0.1, 0.9] | *α_i_*=0.5 |
| **Modality Feature Correlation Threshold** | [0.1, 0.5] | *ϵ*=0.3 |
| **Dynamic Weight Adjustment Step Size** | [0.01, 0.1 | Δ*α*=0.05 |
| **Maximum Iteration Count** | [50, 500] | *T*_max_=200 |
| **Distance Type for Matching Similarity Function** | Euclidean Distance, Cosine Distance | Euclidean Distance |
| **Correlation Matrix Update Frequency** | Every 10 Iterations | Every 10 Iterations |

Based on the content in Table 1, the initial weight value of 0.5 is chosen to balance the influence of all modalities at the initial stage. After experimental adjustments, the dynamic adjustment mechanism could quickly optimize weight allocation, so the initial value does not need to be heavily skewed. The correlation threshold was determined through the cross-correlation matrix calculation. Statistical analysis of the correlation distribution for each modality pair showed that a threshold of 0.3 effectively filters out low-correlation matches. Experiments with different step sizes revealed that a step size of 0.05 provided the best balance between the convergence speed of weight adjustment and matching accuracy. The algorithm typically reaches stable convergence within 200 iterations, so 200 iterations was chosen as the maximum iteration count. A sensitivity analysis was performed on the range from 50 to 500 iterations. After comparing Euclidean distance and cosine distance, Euclidean distance performed better for the numerical features in this task (such as MFCC features and visual keypoints), so it was selected as the default.

Results and Discussion

## Hyperparameter Tuning Results

The process of confirming the hyperparameters for this study is shown in Fig. 2.

**Fig. 2.** Hyperparameter Setting Results (a: Initial Weight Value and Correlation Threshold; b: Adjustment Step Size)

In Fig. 2, when *α_i_*=0.5, the matching accuracy and information integration efficiency reached a high level, while the user satisfaction score was also optimal and the computational complexity was moderate. Therefore, *α_i_*=0.5 was selected as the default initial value. When *ϵ*=0.3, all metrics performed best, with optimal matching accuracy and user satisfaction scores, while the computational complexity remained moderate. Thus, *ϵ*=0.3 was chosen as the final threshold. When Δ*α*=0.05, matching accuracy was the highest, the convergence iteration count was low, and both information integration efficiency and user satisfaction were optimal. Therefore, Δ*α*=0.05 was selected. Considering the balance between accuracy and runtime, *T_max_*=200 was chosen as the default value. Based on the experimental results, Euclidean distance was selected as the default distance metric.

## Matching Efficiency and Accuracy

To assess the performance of the improved Kuhn-Munkres algorithm in multimodal information matching tasks, this study compared its matching accuracy and information integration efficiency with five benchmark methods. These methods include the traditional Kuhn-Munkres algorithm, DeepCCA, MMTF, GMN, and CAT. These benchmark methods represent mainstream techniques in the current multimodal matching field. They cover a range of paradigms, from traditional algorithms to deep learning approaches. The experimental data were based on the CMU-MOSI and IEMOCAP datasets, and the test tasks included speech-visual, speech-text, and full-modal matching tasks. The performance of each method in terms of matching accuracy and information integration efficiency was recorded through multiple rounds of experiments. The specific results are shown in Fig. 3:

**Fig. 3.** Comparison of Matching Accuracy and Information Integration Efficiency Across Different Methods

In Fig. 3, the improved Kuhn-Munkres algorithm achieved a matching accuracy of 87.7%, which is a 28.2% improvement over the traditional Kuhn-Munkres algorithm. In comparison with other advanced algorithms, the improved algorithm outperformed DeepCCA, MMTF, GMN, and CAT in matching accuracy. It exceeded them by 15.3%, 12.9%, 9.6%, and 5.8%, respectively. This shows a significant advantage in multimodal matching tasks. In terms of information integration efficiency, the improved algorithm showed an 18.7% improvement, significantly outperforming DeepCCA (8.5%), MMTF (10.2%), GMN (12.7%), and CAT (15.3%). The experimental results indicate that the combination of dynamic weight adjustment and cross-modal correlation constraints significantly enhanced the algorithm’s applicability in complex multimodal combination tasks. In multimodal combination tasks, such as speech-visual or full-modal matching, dynamic weight adjustment of the speech modality further improved matching accuracy. Additionally, enhancing the robustness of the visual modality boosted the system’s integration efficiency. The results show that in full-modal tasks, the improved algorithm’s integration efficiency is 21.3% higher than that of the traditional Kuhn-Munkres algorithm.

## Algorithm Computational Complexity Analysis

To evaluate the improved algorithm’s computational efficiency, this study compared its theoretical complexity and actual running time with five benchmark methods: traditional Kuhn-Munkres, DeepCCA, MMTF, GMN, and CAT. In terms of theoretical complexity, the traditional Kuhn-Munkres algorithm has a complexity of *O*(*n³*). In contrast, the proposed improved algorithm reduces certain operations to *O*(*n²logn*) by introducing dynamic weight adjustment and cross-modal correlation matrix constraints. This optimization effectively reduces computational costs. The experimental running time was measured using multimodal matching tasks. Five datasets of different sizes were tested to assess the algorithm’s runtime efficiency across various dataset scales, as shown in Fig. 4.

**Fig. 4.** Comparison of Computational Complexity and Actual Running Time Across Different Methods

In Fig. 4, the theoretical computational complexity of the improved algorithm was optimized to *O*(*n*^2^log*n*), similar to GMN. However, its joint optimization of dynamic weight adjustment and inter-modality correlation constraints gave it an advantage in actual runtime. The running times of the improved algorithm for sample sizes of 100, 500, and 1000 were 0.58 seconds, 2.89 seconds, and 5.73 seconds, respectively. Compared to the traditional Kuhn-Munkres algorithm, the improved algorithm reduced average runtime by 15.4%, especially demonstrating better computational efficiency in scenarios with larger datasets.

To further analyze the impact of dataset size on algorithm runtime, this study plotted the curve of dataset size versus runtime, as shown in Fig. 5.

**Fig. 5.** Curve of Data Size vs. Running Time

The results in Fig. 5 show that the computation time of the improved algorithm increases more slowly as the dataset size grows. This indicates better scalability compared to traditional methods for large-scale datasets. The running time for the improved algorithm grows at a much slower rate than traditional methods when processing large-scale data. This further validates the effectiveness of the algorithm optimization and its applicability for handling large-scale data.

## User Interaction Satisfaction Survey Results

To evaluate the improvement in user interaction, this study designed multimodal interaction tasks. It also collected user satisfaction scores through a questionnaire survey. The questionnaire scoring range was from 1 to 10, and the experiment recruited 30 volunteers. Each volunteer completed 10 sets of multimodal interaction tasks involving speech, visual, and text modality matching and feedback. Compared with the traditional Kuhn-Munkres algorithm and other benchmark methods, the improved algorithm showed a 19.5% increase in user satisfaction scores. The detailed statistical data are shown in Fig. 6.

**Fig. 6.** User Satisfaction Scores for Different Algorithms

In Fig. 6, the improved algorithm's advantages in feedback speed and matching accuracy were the main reasons for the increased user satisfaction. Its average satisfaction score was 8.9, with a standard deviation of only 0.38, indicating high consistency in the ratings.

Discussion

The dynamic weight adjustment mechanism is a key innovation in this algorithm. Its primary role is to adjust the relative importance of modality features in real time. This addresses the heterogeneity of multimodal data. The mechanism dynamically evaluates the importance of modality features, avoiding the limitations of manually set weights in traditional methods. It also allows the algorithm to adapt to changes in feature distribution across different scenarios. In practical applications, this dynamic optimization capability balances modality differences more effectively. It is particularly useful when the quality of certain modality information is uneven. It also helps when specific modalities are more important. This demonstrates the algorithm’s greater flexibility. The introduction of inter-modality correlation constraints is crucial for addressing the consistency problem in multimodal information integration. Multimodal data typically have inherent semantic correlations; for example, speech and facial expressions often reflect emotions together. By adding inter-modality relationships into the matching process, this constraint ensures the robustness and rationality of the final matching results. Theoretically, this design reduces the interference of low-quality features, enhancing the overall stability of the matching process. In practice, this mechanism significantly improves the algorithm’s adaptability in complex scenarios, especially when multimodal information contains noise or partial missing data, while still achieving consistent performance. The traditional Kuhn-Munkres algorithm faces computational bottlenecks when handling large-scale data due to its high complexity. This study introduces a hierarchical optimization strategy and constraints, effectively reducing the algorithm's computational complexity. This optimization does not merely reduce computation steps, but refines the matching conditions and screening process, narrowing the scope of global search, thus achieving a balance between time efficiency and matching quality. This design not only reduces computational overhead theoretically but also demonstrates efficient support for multimodal tasks in practice, providing a solid foundation for applications requiring real-time performance. In the user interaction experiments, the improved algorithm's feedback speed and stability were the main driving forces behind the increased user satisfaction. The underlying reasons for this can be attributed to two factors: First, the improved algorithm quickly adapts to the dynamic changes of multimodal data, ensuring the system maintains a high response speed when handling complex tasks. Second, the consistency and accuracy of the matching results make users feel more reliable during interactions. This improvement in user experience is not only the result of technical performance but also reflects a deep understanding of user needs in the algorithm's design.

Conclusion

This study addresses the issues of low efficiency, insufficient accuracy, and lack of flexibility in the multimodal information matching process. It proposes an improved Kuhn-Munkres algorithm, which introduces a dynamic weight adjustment mechanism and inter-modality correlation constraints, thereby constructing an information integration model suitable for complex multimodal interaction scenarios. Using the publicly available CMU-MOSI and IEMOCAP datasets, the study systematically evaluates the matching performance, computational complexity, and user interaction satisfaction of the improved algorithm. The results show that the improved algorithm significantly enhances the accuracy and integration efficiency of multimodal matching, demonstrating strong applicability and robustness. The dynamic weight adjustment mechanism can adapt to the varying importance of different modality features, effectively addressing the problem of modality heterogeneity. The modality correlation constraint further ensures inter-modality coherence during the matching process, strengthening the stability of the algorithm. However, there are still some limitations in this study. First, the datasets used in the experiments are limited in terms of modality types and scale; future research could incorporate more diverse multimodal data to extend the applicability of the method. Second, during the feature selection and extraction phase, this study primarily uses traditional feature engineering methods, but future work could combine deep learning techniques to achieve a more automated and dynamic feature extraction process. Additionally, this study mainly focuses on multimodal matching in offline tasks, and future research could explore its performance in real-time online applications. The proposed improved algorithm not only provides an efficient and robust solution for multimodal information matching in theory but also shows great potential for real-world applications.

**Data available statement**

All relevant data are within the manuscript and its Supporting Information files.

References

1. Chen X, Ye P, Huang L, Wang C, Cai Y, Deng L, et al. Exploring science-technology linkages: A deep learning-empowered solution. Information Processing & Management. 2023; 60(2): 103255.
2. Bannis A, Pan S, Ruiz C, Shen J, Noh H Y, Zhang P. IDIoT: Multimodal framework for ubiquitous identification and assignment of human-carried wearable devices. ACM Transactions on Internet of Things. 2023; 4(2): 1-25.
3. Qiu Z, Xie Z, Ji Z, Mao Y, Cheng K. HGSMAP: a novel heterogeneous graph-based associative percept framework for scenario-based optimal model assignment. Knowledge and Information Systems. 2024; 2024: 1-38.
4. Zha Y, Shangguan W, Chai L, Chen J. Hierarchical Perception Enhancement for Different Levels of Autonomous Driving: A Review. IEEE Sensors Journal. 2024; 2024: 1-12.
5. Müller M, Dupuis A, Zeulner T, Vazquez I, Hagerer J, Gloor P A. Predicting Team Well-Being through Face Video Analysis with AI. Applied Sciences. 2024; 14(3): 1284.
6. Liu X, Luo Y, Yan K, Chen J, Lei Z. CMC2R: Cross‐modal collaborative contextual representation for RGBT tracking. IET Image Processing. 2022; 16(5): 1500-1510.
7. Xie L, Misra S, Suresh N, Soza-Soto J, Furuhata T, Shimada K. Learning 3D human–object interaction graphs from transferable context knowledge for construction monitoring. Computers in Industry. 2025; 164: 104171.
8. Alqudsi Y, Makaraci M. Exploring advancements and emerging trends in robotic swarm coordination and control of swarm flying robots: A review. Proceedings of the Institution of Mechanical Engineers, Part C: Journal of Mechanical Engineering Science. 2025; 239(1): 180-204.
9. Liu Y, Xie B, Xu G, Zhao J, Li T. A two-stage dispatching approach for one-to-many ride-sharing with sliding time windows. Neural Computing and Applications. 2024; 2024: 1-27.
10. Bruglieri M, Pisacane O. A survey on emergent trends in the optimization of car‐sharing systems. International Transactions in Operational Research. 2023; 30(6): 2867-2908.
11. Yang E, Milisav F, Kopal J, Holmes A J, Mitsis G D, Misic B, et al. The default network dominates neural responses to evolving movie stories. Nature communications. 2023; 14(1): 4197.
12. Wang S, Yan R, Huang P, Dai G, Song Y, Shu X. Com-stal: Compositional spatio-temporal action localization. IEEE Transactions on Circuits and Systems for Video Technology. 2023; 33(12): 7645-7657.
13. Gao J, Li P, Laghari A A, Srivastava G, Gadekallu T R, Abbas S, Zhang J. Incomplete multiview clustering via semidiscrete optimal transport for multimedia data mining in IoT. ACM Transactions on Multimedia Computing, Communications and Applications. 2024; 20(6): 1-20.
14. Feng S, Duan P, Ke J, Yang H. Coordinating ride-sourcing and public transport services with a reinforcement learning approach. Transportation Research Part C: Emerging Technologies. 2022; 138: 103611.
15. Telikani A, Sarkar A, Du B, Santoso F, Shen J, Yan J, et al. Unmanned Aerial Vehicle-Aided Intelligent Transportation Systems: Vision, Challenges, and Opportunities. IEEE Communications Surveys & Tutorials. 2025; 2025: 1-11.
16. Russo M, Giugliano A, Flenghi G, Carnevali L, Martone M, Marrella A, et al. Augmented Geometry in university education. Inted Proceedings. 2023; 2023: 1787-1796.
17. Farkaš I. Transforming Cognition and Human Society in the Digital Age. Biological Theory. 2024; 2024: 1-13.
18. Chen X, Xie H, Tao X, Xu L, Wang J, Dai H N, et al. A topic modeling‐based bibliometric exploration of automatic summarization research. Wiley Interdisciplinary Reviews: Data Mining and Knowledge Discovery. 2024; 2024: e1540.
19. Tang Z, Gao Y, Hui T, Peng F, Liu S. MI3C: Mining intra-and inter-image context for person search. Pattern Recognition. 2024; 148; 110169.
20. Wang R, Tang Z, Zhou Q, Liu X, Hui T, Tan Q, et al. Unified transformer with isomorphic branches for natural language tracking. IEEE Transactions on Circuits and Systems for Video Technology. 2023; 33(9): 4529-4541.
21. Le N, Do T, Do K, Nguyen H, Tjiputra E, Tran Q D, et al. Controllable group choreography using contrastive diffusion. ACM Transactions on Graphics (TOG). 2023; 42(6): 1-14.
22. Zhu J, Guha A, Do D, Xu M, Nguyen X, Zhao D. Functional optimal transport: regularized map estimation and domain adaptation for functional data. Journal of Machine Learning Research. 2024; 25(276): 1-49.
23. Sumitkumar R, Al-Sumaiti A S. Shared autonomous electric vehicle: Towards social economy of energy and mobility from power-transportation nexus perspective. Renewable and Sustainable Energy Reviews. 2024; 197: 114381.
24. Zeng Y, Yin R, Luo M, Chen J, Pan Z, Lu Y, et al. Identifying spatial domain by adapting transcriptomics with histology through contrastive learning. Briefings in Bioinformatics. 2023; 24(2): bbad048.
25. Senel N, Kefferpütz K, Doycheva K, Elger G. Multi-sensor data fusion for real-time multi-object tracking. Processes. 2023; 11(2): 501.
26. El Natour G, Bresson G, Trichet R. Multi-Sensors System and Deep Learning Models for Object Tracking. Sensors. 2023; 23(18): 7804.
27. Martí R, Sevaux M, Sörensen K. Fifty years of metaheuristics. European journal of operational research-Amsterdam. 2025; 321(2): 345-362.
28. Sharma S, Kumar V. A comprehensive review on multi-objective optimization techniques: Past, present and future. Archives of Computational Methods in Engineering. 2022; 29(7): 5605-5633.
29. Monai E, Pini L, Palacino F, Bisio M, Bernocchi F, Salvalaggio A, et al. Convergence of visual and motor awareness in human parietal cortex. Annals of Neurology. 2024; 95(1): 90-103.
30. Chen B, Xu S, Xu H, Bian X, Guo N, Xu X, et al. Structural deep multi-view clustering with integrated abstraction and detail. Neural Networks. 2024; 175: 106287.
