# Supplementary material for: Structured matching models in multimodal information fusion: An optimized Kuhn-Munkres algorithm
Source: PLoS One. 2025 Nov 21;20(11):e0326662. doi: 10.1371/journal.pone.0326662 (PMC12637911; doi:10.1371/journal.pone.0326662)
Supplement: Attachment — Submitted filename: Response to Reviewers.docx [file pone.0326662.s005.docx]

The reviewers have identified several critical areas requiring improvement:

1. Technical details: Please provide sample size clarification, replication details, and more thorough explanation of your algorithm's mechanisms.

Reply: Your suggestion is very good. We have added more explanations about the research details in the third section. Before each round of matching task starts, the system calculates the variance of each modality feature in turn (as the basis for importance scoring) based on the feature set extracted from the current input sample batch, and generates the modality weight vector *α* = {*α*₁, *α*₂, ..., *α_k_*} of the current round through normalization. Then, for each pair of candidate matches (*u*,*v*) in the bipartite graph, the similarity *f_i_*(*u,v*) under each modality is calculated respectively, and finally the weighted edge weight is obtained. We have added explanations about sample size and replication details in the second section of the method section. To ensure that the data fully covers different modal performances, this paper selects 500 annotated video clips from the CMU-MOSI dataset as training and test samples; 600 complete dialogue rounds are selected from IEMOCAP, including samples with different gender and emotion labels. During the experiment, all tasks are set up with 5 independent repeated experiments, and the weights and order are initialized with different random seeds to control the sensitivity of the model to the initial state. All results are reported as mean values with standard deviations to describe variability and ensure the replicability and robustness of the experiments.

2. Statistical analysis: Include appropriate statistical tests with p-values, standard deviations, confidence intervals, and error bars in figures.

Reply: Your comments are very objective. We have added these statistical analyses to the results section and modified the images in the results section. The average accuracy of the improved algorithm is 87.7%, with a standard deviation of ±0.53, which is significantly higher than all the baseline methods. Compared with the CAT method, the difference is statistically significant (t = 6.14, p = 0.002), and the effect size Cohen’s d = 1.54 is a large effect level. Compared with the traditional Kuhn-Munkres algorithm, the improvement in matching accuracy is as high as 3.58, indicating that the improved algorithm is not only statistically significant, but also has a substantial difference.

3. Theoretical framework: More explicitly state your theoretical contribution, include an ablation study, and better highlight the research gap.

Reply: Your suggestion is very good. We added a supplement about the theoretical framework in the first section of the method section. The theoretical innovation of this paper is reflected in: based on graph matching, it integrates feature importance modeling and inter-modal structural constraints. A multimodal information matching framework with both interpretability and practicality is proposed. This framework not only provides theoretical support for subsequent algorithm implementation, but also provides a modeling reference for subsequent task matching in multimodal scenarios.

We added ablation experiments in the results section. This paper designs the following 4 models for ablation experiments: A: full model (complete improved algorithm), specifically including dynamic weights + correlation constraints (this paper's model); B: remove dynamic weights, including fixed weights + correlation constraints; C: remove correlation constraints, including dynamic weights + no correlation matrix; D: remove all improvements, including fixed weights + no correlation (degenerated to traditional KM). Model A achieved the best performance in all indicators, with a matching accuracy of 87.7%, an information integration efficiency of 84.9%, a satisfaction score of 8.9, and a stable running time; after removing the dynamic weight mechanism (model B), the accuracy dropped to 81.3%, and the satisfaction score dropped by 0.8 points; removing the correlation constraint (model C) also showed a performance decline, but the degree was slightly less than that of B, indicating that the dynamic weight had a greater impact on the matching accuracy; Model D had the worst performance, and all indicators were significantly lower than the improved model (p<0.01), verifying the necessity of the two modules. In summary, the ablation experiment results show that the dynamic weight mechanism and the inter-modal correlation constraint have a significant and complementary role in improving the performance of the algorithm. The combination of the two can achieve comprehensive optimization of accuracy, efficiency, and experience.

4. Presentation quality: Improve figure resolution and clarity, provide formal algorithm complexity analysis, and enhance language quality throughout.

Reply: Thanks for your reminder. We have optimized the images in the full text. We added an analysis of the algorithm complexity in the results section. First, in the process of constructing the multimodal graph, the algorithm needs to calculate the modal feature similarity of all node pairs and perform weighted fusion. Since the number of modalities is fixed, the overall computational workload is mainly determined by the number of node pairs, which is a typical quadratic overhead. Second, the dynamic weight mechanism updates the importance score of each modality at a fixed period, which is less time-consuming to calculate and has a negligible overall impact. Third, before matching, the inter-modal correlation matrix is introduced as a constraint to screen potential matching paths. This step further improves the rationality of matching, but it is a lightweight operation in terms of computation. Finally, the core matching part is structurally optimized based on the original Kuhn-Munkres algorithm, reducing it from the traditional cubic level to the approximate square logarithmic level, significantly reducing the time consumption under big data conditions. We have improved the language quality of the full text.

Please submit your revised manuscript by May 08 2025 11:59PM. If you will need more time than this to complete your revisions, please reply to this message or contact the journal office at [plosone@plos.org](mailto:plosone@plos.org). When you're ready to submit your revision, log on to https://www.editorialmanager.com/pone/ and select the 'Submissions Needing Revision' folder to locate your manuscript file.

Please include the following items when submitting your revised manuscript:

- A rebuttal letter that responds to each point raised by the academic editor and reviewer(s). You should upload this letter as a separate file labeled 'Response to Reviewers'.
- A marked-up copy of your manuscript that highlights changes made to the original version. You should upload this as a separate file labeled 'Revised Manuscript with Track Changes'.
- An unmarked version of your revised paper without tracked changes. You should upload this as a separate file labeled 'Manuscript'.

If you would like to make changes to your financial disclosure, please include your updated statement in your cover letter. Guidelines for resubmitting your figure files are available below the reviewer comments at the end of this letter.

If applicable, we recommend that you deposit your laboratory protocols in protocols.io to enhance the reproducibility of your results. Protocols.io assigns your protocol its own identifier (DOI) so that it can be cited independently in the future. For instructions see: <https://journals.plos.org/plosone/s/submission-guidelines#loc-laboratory-protocols>. Additionally, PLOS ONE offers an option for publishing peer-reviewed Lab Protocol articles, which describe protocols hosted on protocols.io. Read more information on sharing protocols at <https://plos.org/protocols?utm_medium=editorial-email&utm_source=authorletters&utm_campaign=protocols>.

We look forward to receiving your revised manuscript.

Kind regards,

Yosi Kristian
Academic Editor
PLOS ONE

Journal requirements:

When submitting your revision, we need you to address these additional requirements.

1. Please ensure that your manuscript meets PLOS ONE's style requirements, including those for file naming. The PLOS ONE style templates can be found at

https://journals.plos.org/plosone/s/file?id=wjVg/PLOSOne_formatting_sample_main_body.pdf and

<https://journals.plos.org/plosone/s/file?id=ba62/PLOSOne_formatting_sample_title_authors_affiliations.pdf>

2. Please note that PLOS ONE has specific guidelines on code sharing for submissions in which author-generated code underpins the findings in the manuscript. In these cases, we expect all author-generated code to be made available without restrictions upon publication of the work. Please review our guidelines at https://journals.plos.org/plosone/s/materials-and-software-sharing#loc-sharing-code and ensure that your code is shared in a way that follows best practice and facilitates reproducibility and reuse.

3. PLOS requires an ORCID iD for the corresponding author in Editorial Manager on papers submitted after December 6th, 2016. Please ensure that you have an ORCID iD and that it is validated in Editorial Manager. To do this, go to ‘Update my Information’ (in the upper left-hand corner of the main menu), and click on the Fetch/Validate link next to the ORCID field. This will take you to the ORCID site and allow you to create a new iD or authenticate a pre-existing iD in Editorial Manager.

4. Please include captions for your Supporting Information files at the end of your manuscript, and update any in-text citations to match accordingly. Please see our Supporting Information guidelines for more information: http://journals.plos.org/plosone/s/supporting-information.

Additional Editor Comments:

The consensus from the three reviewers indicates several significant issues that need addressing:

Technical soundness issues:
Sample size and replication details are inadequate
The specific mechanisms of the improved Kuhn-Munkres algorithm aren't sufficiently detailed
The paper lacks crucial explanations to support its findings
Reply: Your suggestion is good. We have added explanations about sample size and replication details in the second subsection of the Methods section. To ensure that the data fully covers different modal performances, this paper selected 500 annotated video clips from the CMU-MOSI dataset as training and test samples; and selected 600 complete dialogue turns from the IEMOCAP dataset, including samples with different gender and emotion labels. During the experiment, all tasks were set up for 5 independent replications, and the weights and orders were initialized with different random seeds to control the sensitivity of the model to the initial state. All results are reported as averages with standard deviations to describe variability to ensure the replicability and robustness of the experiment. We have added more explanations about the research details in the third subsection of the Methods section. Before each round of matching task starts, the system calculates the variance of each modality feature in turn (as the basis for importance scoring) based on the feature set extracted from the current input sample batch, and generates the modality weight vector *α* = {*α*₁, *α*₂, ..., *α_k_*} of the current round through normalization. Then, for each pair of candidate matches (*u*,*v*) in the bipartite graph, the similarity *f_i_*(*u,v*) under each modality is calculated respectively, and finally the weighted edge weight is obtained. In addition, we added more supplements and explanations in the results section.

Statistical rigor concerns:
Need for appropriate statistical tests with p-values
Missing standard deviations or confidence intervals
Lack of error bars in figures
Incomplete cross-validation procedure explanation
Reply: This is a good suggestion. We have added statistical analysis to the Results section and modified the figures, adding error bars to some of the figures. The average accuracy of the improved algorithm is 87.7%, with a standard deviation of ±0.53, which is significantly higher than all the baseline methods. Compared with the CAT method, the difference is statistically significant (t = 6.14, p = 0.002), and the effect size Cohen’s d = 1.54 is a large effect level. Compared with the traditional Kuhn-Munkres algorithm, the improvement in matching accuracy is as high as 3.58, indicating that the improved algorithm is not only statistically significant but also has a substantial difference.

We have added a description of the crossover procedure in the Results section. To ensure the robustness of the results, all performance tests use 5-fold cross-validation, with random division of the training set and validation set in each round. The accuracy, running time, and information integration efficiency of each method on each fold are recorded independently, and the final results report the average and standard deviation.

Novelty and contribution issues:
Theoretical framework needs clearer articulation
An ablation study is needed to demonstrate the contribution of each component
The research gap should be better highlighted in introduction and related work
Reply: Thank you for your suggestion. We added the content about theoretical framework in the first section of the method section. The theoretical innovation of this paper is reflected in: based on graph matching, it integrates feature importance modeling and inter-modal structural constraints. A multimodal information matching framework with both interpretability and practicality is proposed. This framework not only provides theoretical support for subsequent algorithm implementation, but also provides a model reference for subsequent task matching in multimodal scenarios.

We added ablation experiments in the results section. This paper designs the following 4 models for ablation experiments: A: full model (complete improved algorithm), specifically including dynamic weights + correlation constraints (this paper's model); B: remove dynamic weights, including fixed weights + correlation constraints; C: remove correlation constraints, including dynamic weights + no correlation matrix; D: remove all improvements, including fixed weights + no correlation (degenerated to traditional KM). Model A achieved the best performance in all indicators, with a matching accuracy of 87.7%, an information integration efficiency of 84.9%, a satisfaction score of 8.9, and a stable running time; after removing the dynamic weight mechanism (model B), the accuracy dropped to 81.3%, and the satisfaction score dropped by 0.8 points; removing the correlation constraint (model C) also showed a performance decline, but the degree was slightly less than that of B, indicating that the dynamic weight had a greater impact on the matching accuracy; Model D had the worst performance, and all indicators were significantly lower than the improved model (p<0.01), verifying the necessity of the two modules. In summary, the ablation experiment results show that the dynamic weight mechanism and the inter-modal correlation constraint have a significant and complementary role in improving the performance of the algorithm. The combination of the two can achieve comprehensive optimization of accuracy, efficiency and experience.

We have supplemented this part in the introduction and literature review. However, although existing studies have proposed a variety of fusion strategies, most methods still remain at the level of static feature fusion, ignoring the matching complexity caused by the dynamic changes in semantics and differences in weight distribution between modalities. In addition, the current mainstream methods lack clear structural optimization strategies when modeling the collaborative relationship between modalities, resulting in limited overall fusion effects. Therefore, there is an urgent need for a unified model that can theoretically solve both the dynamic changes in modal weights and structural matching to bridge this technical gap.

Presentation quality problems:
Figure quality is poor (blurred and unclear images)
Language needs improvement (paragraph structure, clarity)
Formal algorithm complexity analysis is missing
Reply: Your suggestion is good. We have optimized the images throughout the article. We have optimized the clarity of the paragraphs throughout the article. We added more explanation about the research details in the third subsection of the methods section. Before each round of matching task starts, the system calculates the variance of each modality feature in turn (as the basis for importance scoring) based on the feature set extracted from the current input sample batch, and generates the modality weight vector *α* = {*α*₁, *α*₂, ..., *α_k_*} of the current round through normalization. Then, for each pair of candidate matches (*u*,*v*) in the bipartite graph, the similarity *f_i_*(*u,v*) under each modality is calculated respectively, and finally the weighted edge weight is obtained.

5. Review Comments to the Author

Please use the space provided to explain your answers to the questions above. You may also include additional comments for the author, including concerns about dual publication, research ethics, or publication ethics. (Please upload your review as an attachment if it exceeds 20,000 characters)

Reviewer #1: Some parts need to be organized and recycled in several paragraphs. Language needs more attention, in one paragraph there are at least 3 sentences. please add a few more sentences. The improved Kuhn-Munkres algorithm is described in general terms (such as the use of dynamic weights and correlation matrices), but the specific mechanism of how this improvement works is not described in sufficient detail.

Reply: Thank you for your reminder, we have optimized the structure of the article. We added more explanations about the research details in the third subsection of the methods section. Before each round of matching task starts, the system calculates the variance of each modality feature in turn (as the basis for importance scoring) based on the feature set extracted from the current input sample batch, and generates the modality weight vector *α* = {*α*₁, *α*₂, ..., *α_k_*} of the current round through normalization. Then, for each pair of candidate matches (*u*,*v*) in the bipartite graph, the similarity *f_i_*(*u,v*) under each modality is calculated respectively, and finally the weighted edge weight is obtained.

Reviewer #2: This manuscript presents significant improvements to the Kuhn-Munkres algorithm for multimodal information integration in human-computer interaction. Below is a summary of strengths and required improvements to meet PLOS ONE standards:
Strengths

Clear methodological approach using established datasets (CMU-MOSI and IEMOCAP)
Innovative modifications to the Kuhn-Munkres algorithm (dynamic weight adjustment and cross-modal correlation constraints)
Comparative analysis against five baseline methods with quantitative results
Promising performance improvements (28.2% in matching accuracy, 18.7% in efficiency, 15.4% in computation time)
User experience validation (19.5% improvement in satisfaction ratings)
Reply: Thank you for your affirmation.
Required Improvements for Publication
Technical Soundness

Sample size clarification: Specify the number of test cases used from each dataset
Replication details: Indicate whether experiments were replicated and how variability was managed
Reply: Thank you for your suggestion. We have added a description of sample size and variability in the second subsection of the methods section. To ensure that the data fully covers different modal performances, this paper selected 500 annotated video clips from the CMU-MOSI dataset as training and test samples; 600 complete dialogue turns were selected from IEMOCAP, including samples with different gender and emotion labels. During the experiment, all tasks were set up with 5 independent repeated experiments, and the weights and order were initialized with different random seeds to control the sensitivity of the model to the initial state. All results report the mean value with standard deviation to describe the variability to ensure the replicability and robustness of the experiment.

Statistical Rigor

Statistical significance testing: Include appropriate statistical tests (t-tests or ANOVA) with p-values
Variability measurements: Add standard deviations or confidence intervals for all performance metrics
Error bars: Include these in figures to visualize variability
Cross-validation procedure: Explain the validation methodology (e.g., k-fold) and report performance across folds
Effect size analysis: Calculate and report effect sizes to contextualize improvements
Reply: Thank you for your reminder. We made significant modifications in the results section, including changes to the images, addition of statistical analysis related content, and explanation of the effect size. The average accuracy of the improved algorithm is 87.7%, with a standard deviation of ± 0.53, significantly higher than all benchmark methods. Compared with the CAT method, the difference is statistically significant (t=6.14, p=0.002), and the effect size Cohen's d=1.54, belonging to the large effect level. Compared with the traditional Kuhn Munkres algorithm, the improvement effect of matching accuracy is as high as 3.58, indicating that the improved algorithm is not only statistically significant, but also has substantial differences.

We have added explanations for the crossover program in the results section. To ensure the robustness of the results, all performance tests were conducted using 5-fold cross validation, with each round randomly divided into a training set and a validation set. Independently record the accuracy, running time, and information integration efficiency of each method at each fold, and report the average and standard deviation of the final results.

Novelty Enhancement

Theoretical contribution: More explicitly state the theoretical framework advanced by this research
Ablation study: Demonstrate the relative contribution of each proposed component
Comparative discussion: Expand on fundamental differences from existing state-of-the-art methods
Reply: Your suggestion is very good. We added a supplement on the theoretical framework in the first subsection of the methods section. The theoretical innovation of this article is reflected in the fusion of feature importance modeling and modal structural constraints based on graph matching. A multimodal information matching framework that combines interpretability and practicality has been proposed. This framework not only provides theoretical support for subsequent algorithm implementation, but also provides a modeling reference for task matching in multimodal scenarios.

We have added ablation experiments in the results section. This article designs the following four models for ablation experiments: A: Full model (complete improved algorithm), including dynamic weights and correlation constraints (this paper's model); B: Remove dynamic weights, including fixed weights and correlation constraints; C: Remove correlation constraints, including dynamic weights and uncorrelated matrices; D: Remove all improvements, including fixed weights and no correlation (degraded to traditional KM). Model A achieved the best performance in all indicators, with a matching accuracy of 87.7%, information integration efficiency of 84.9%, satisfaction score of 8.9, and stable running time; After removing the dynamic weight mechanism (Model B), the accuracy decreased to 81.3% and the satisfaction score decreased by 0.8 points; Removing the correlation constraint (Model C) also resulted in performance degradation, but to a slightly smaller extent than Model B, indicating that dynamic weights have a greater impact on matching accuracy; The performance of Model D is the worst, with significantly lower indicators than the improved model (p<0.01), which verifies the necessity of the two modules. Overall, the ablation experiment results indicate that the dynamic weight mechanism and inter modal correlation constraints have significant and complementary effects in improving algorithm performance. The combination of the two can achieve comprehensive optimization of accuracy, efficiency, and user experience.

We have optimized the discussion section, and compared with mainstream advanced algorithms, our method in this paper has essential differences in matching logic. Currently, CAT adopts a cross modal attention mechanism to capture semantic correlations, while this study explicitly models the inter modal correlation matrix and introduces structural constraints to optimize the matching path from the bottom layer.

Visual and Presentation Quality

Figure quality: Improve resolution and clarity of all figures
Algorithm complexity analysis: Provide more formal and rigorous complexity analysis
Reply: What a great suggestion. We have enhanced the image quality of the entire text.

We have added an analysis of algorithm complexity in the results section. Firstly, in the process of constructing multimodal graphs, the algorithm needs to calculate the similarity of modal features for all node pairs and perform weighted fusion. Due to the fixed number of modes, the overall computational complexity is mainly determined by the number of node pairs, which is a typical square level overhead. Secondly, the dynamic weighting mechanism updates the importance scores of each modality at a fixed period, which is computationally efficient and has a negligible overall impact. Thirdly, introducing the inter modal correlation matrix as a constraint condition before matching to screen potential matching paths further enhances the rationality of matching, but it is a lightweight operation in terms of computation. Finally, the core matching part was structurally optimized based on the original Kuhn Munkres algorithm, reducing it from traditional cubic level to approximately square logarithmic level, significantly reducing time consumption under big data conditions.

Conclusion
This manuscript shows promise for publication in PLOS ONE with the implementation of the suggested improvements. The novel approach to multimodal information integration demonstrates practical significance, but requires enhanced statistical rigor and clearer articulation of theoretical contributions to fully meet publication standards.

Reply: Thank you for the reminder. We have added more content about statistical analysis in the results section. The average accuracy of the improved algorithm is 87.7%, with a standard deviation of ± 0.53, significantly higher than all benchmark methods. Compared with the CAT method, the difference is statistically significant (t=6.14, p=0.002), and the effect size Cohen's d=1.54, belonging to the large effect level. Compared with the traditional Kuhn Munkres algorithm, the improvement effect of matching accuracy is as high as 3.58, indicating that the improved algorithm is not only statistically significant, but also has substantial differences.

We added theoretical contributions in the introduction and a supplement on the theoretical framework in the first subsection of the methods section. The theoretical innovation of this article is reflected in the fusion of feature importance modeling and modal structural constraints based on graph matching. A multimodal information matching framework that combines interpretability and practicality has been proposed. This framework not only provides theoretical support for subsequent algorithm implementation, but also provides a modeling reference for task matching in multimodal scenarios.

Reviewer #3: The manuscript discusses the challenge of integrating heterogeneous data from speech, vision, and text in multimodal HCI systems. The aim is to improve accuracy, efficiency, and user experience in multimodal information integration tasks. The idea is interesting. However, the manuscript is not sufficient enough to be published in this journal as it lacks a lot of crucial explanations to support the findings.
The authors should also include theoretical analysis to better convey methodological improvements, include ablation studies, highlight the research gap in the introduction and related works, and provide more detailed reasoning to support the contribution with deep discussions.
The title also needs to be rewritten to better convey the proposed framework and the images are blurred and unclear.

Reply: Your suggestion is very good. We have supplemented the research differences in the introduction and literature review, and added a supplement on the theoretical framework in the first subsection of the methods section. The theoretical innovation of this article is reflected in the fusion of feature importance modeling and modal structural constraints based on graph matching. A multimodal information matching framework that combines interpretability and practicality has been proposed. This framework not only provides theoretical support for subsequent algorithm implementation, but also provides a modeling reference for task matching in multimodal scenarios.

We have added ablation experiments in the results section. This article designs the following four models for ablation experiments: A: Full model (complete improved algorithm), including dynamic weights and correlation constraints (this paper's model); B: Remove dynamic weights, including fixed weights and correlation constraints; C: Remove correlation constraints, including dynamic weights and uncorrelated matrices; D: Remove all improvements, including fixed weights and no correlation (degraded to traditional KM). Model A achieved the best performance in all indicators, with a matching accuracy of 87.7%, information integration efficiency of 84.9%, satisfaction score of 8.9, and stable running time; After removing the dynamic weight mechanism (Model B), the accuracy decreased to 81.3% and the satisfaction score decreased by 0.8 points; Removing the correlation constraint (Model C) also resulted in performance degradation, but to a slightly smaller extent than Model B, indicating that dynamic weights have a greater impact on matching accuracy; The performance of Model D is the worst, with significantly lower indicators than the improved model (p<0.01), which verifies the necessity of the two modules. Overall, the ablation experiment results indicate that the dynamic weight mechanism and inter modal correlation constraints have significant and complementary effects in improving algorithm performance. The combination of the two can achieve comprehensive optimization of accuracy, efficiency, and user experience.

We have rewritten the article title: Structured Matching Models in Multimodal Information Fusion: An Optimized Kuhn-Munkres Algorithm. We have optimized the images throughout the article.

We highlighted the research gap in the introduction and literature review. However, despite various fusion strategies proposed in previous studies, most methods still remain at the level of static feature fusion, ignoring the matching complexity caused by semantic dynamic changes and weight distribution differences between modalities. In addition, the current mainstream methods lack clear structural optimization strategies when modeling the collaborative relationships between modalities, which limits the overall fusion effect. Therefore, there is an urgent need for a unified model that can theoretically solve the dynamic change of modal weight and structural matching at the same time to bridge this technical gap.
